# Deubiquitinase USP28 inhibits ubiquitin ligase KLHL2-mediated uridine-cytidine kinase 1 degradation and confers sensitivity to 5'-azacytidine-resistant human leukemia cells

**DOI:** 10.7150/thno.36503

**Published:** 2020-01-01

**Authors:** Heng Zhang, He Huang, Xing Feng, Huiwen Song, Zhiyong Zhang, Aizong Shen, Xingfeng Qiu

**Affiliations:** 1 Department of Histology and Embryology, Xiang Ya School of Medicine, Central South University, Changsha, Hunan, China; 2 The affiliated Hospital of Guilin Medical University, Guangxi Key Laboratory of Brain and Cognitive Neuroscience, Guangxi Neurological Diseases Clinical Research Center, Guilin, Guangxi, China; 3 Department of Cardiology, Jiading District Central Hospital Affiliated Shanghai University of Medicine & Health Sciences, Shanghai, China; 4 National Center for International Research of Biological Targeting Diagnosis and Therapy (Guangxi Key Laboratory of Biological Targeting Diagnosis and Therapy Research), Guangxi Medical University, Nanning, China; 5 Department of Surgery, Robert-Wood-Johnson Medical School University Hospital, The State University of New Jersey, New Brunswick, New Jersey, USA; 6 Pharmacy Department, The First Affiliated Hospital of USTC, Division of Life Sciences and Medicine, University of Science and Technology of China, Hefei, Anhui, China; 7 Department of Gastrointestinal Surgery, Zhongshan Hospital of Xiamen University, Xiamen, Fujian, China

**Keywords:** 5'-AZA, UCK1, USP28, KLHL2, ubiquitination

## Abstract

Resistance to the chemotherapeutic drug 5'-azacytidine (5'-AZA) is a major obstacle in the treatment of patients with acute myeloid leukemia (AML). The uridine-cytidine kinase 1 (UCK1) has an established role in activating 5'-AZA and its protein level is significantly downregulated in patients resistant to the drug. However, the underlying molecular mechanism for the reduced UCK1 expression remains to be elucidated.

**Methods:** Using mass spectrometry and molecular biochemistry analyses, we identified specific enzymes mediating UCK1 degradation. Human AML cell lines and murine AML model were used to characterize the effects of these enzymes on 5'-AZA resistance.

**Results:** We demonstrated that the ubiquitin E3 ligase KLHL2 interacted with UCK1 and mediated its polyubiquitination at the K81 residue and degradation. We showed that deubiquitinase USP28 antagonized KLHL2-mediated polyubiquitylation of UCK1. We also provided evidence that ATM-mediated phosphorylation of USP28 resulted in its disassociation from KLHL2 and UCK1 destabilization. Conversely, UCK1 phosphorylation by 5'-AZA-activated ATM enhanced the KLHL2-UCK1 complex formation. Importantly, silencing KLHL2 or USP28 overexpression not only inhibited AML cell proliferation but also sensitized AML cells to 5'-AZA-induced apoptosis *in vitro* and *in vivo*. These results were no longer observed in USP28-deficient cells.

**Conclusions:** Our study revealed a novel mechanism by which the KLHL2/USP28/ATM axis mediates resistance of AML cells to 5'-AZA by regulating UCK1 ubiquitination and phosphorylation. These results have direct clinical implications and provide a rationale for the combination drug treatment of AML patients.

## Introduction

Although approximately 50% of patients with acute myeloid leukemia (AML) show a moderate response to first-line treatment with 5'-azacytidine (5'-AZA), a hypomethylating agent, clinical resistance in patients ultimately leads to poor treatment outcome 1, 2. Therefore, there is an urgent need to uncover the underlying mechanisms of primary resistance to 5'-AZA for therapy improvement.

The mechanisms of 5'-AZA resistance are primarily related to alterations in metabolizing enzymes 3. The uridine/cytidine kinase (UCK) family plays a significant role in the activation and metabolism of 5'-AZA 4. Recent studies showed frequently lower expression of UCK1 found in AML patients without a response to 5'-AZA and silencing UCK1 caused a blunted response to 5'-AZA *in vitro* 5. Hence, the low level of UCK1 might be an important candidate biomarker to predict the efficacy of 5'-AZA treatment. However, the underlying pathogenic mechanisms of UCK1 downregulation were unclear. Given that neither the aberrant methylation of the UCK1 promoter nor polymorphic loci found in the coding sequence of UCK1 were significantly associated with the expression of UCK1 gene or 5'-AZA response 5, we hypothesized that the level of UCK1 protein might be modulated by the ubiquitin-proteasome system 6. Therefore, identification of the enzymes mediating UCK1 ubiquitination and deubiquitination may provide insights helpful in predicting the sensitivity of leukemic cells to 5'-AZA.

Ubiquitination (Ub) is a reversible and dynamic post-translational protein modification 7. The ubiquitin cascade requires E1 ubiquitin-activating, E2 ubiquitin-conjugating, and E3 ligase 8. E3 ligases are responsible for the stability or activity of target proteins. Although Ub is usually diversified based on the generation of chains such as seven internal Lys (K) residues of ubiquitin (Lys6, Lys11, Lys27, Lys29, Lys33, Lys48 or Lys63), only Lys48- and Lys63-linked chains have been well studied 9. Generally, K48-linked polyubiquitin targets proteins for proteasomal degradation, while K63-linked polyubiquitin is thought to mainly regulate intracellular signaling cascades and provide a scaffold for the assembly of protein complexes 7. On the contrary, deubiquitinating enzymes (DUB) mediate the removal of ubiquitin and contain five families: ovarian tumour proteases (OTUs), ubiquitin C-terminal hydrolases (UCHs), Josephins, ubiquitin-specific proteases (USPs), and JAB1/MPN/MOV34 metalloenzymes (JAMM/MPN+). The JAMM/MPN+ family members are zinc metalloproteases while other four families are Cys protease 7. In mammalian cells, there are more than 600 E3 enzymes and about 100 DUBs, and many of them are implicated in tumorigenesis as tumor suppressors or oncogenes 10. Due to their wide-ranging involvement in key signal pathways, these E3 ligase enzymes and DUBs have been an area of intense research and may provide new attractive targets for drug therapy. Thus, it is evident that identifying and understanding the molecular mechanism of E3 ligases and DUBs are of paramount importance for tumor treatment.

In this study, we used an affinity purification and tandem mass spectrometry to identify KLHL2 and USP28 as an E3 ubiquitin ligase and deubiquitinase (DUB) respectively, which antagonistically regulate UCK1 ubiquitination in AML cells and may represent a novel mechanism for relapse or refractoriness to 5'-AZA treatment.

## Methods

### Cell culture

HEK293T, human AML cell line MV4-11 and HL-60 were purchased from the American Type Culture Collection (CRL-3216, CRL-9591, CCL-240). R-HL-60 (5'-AZA resistant cells) were incubated and kept in 3 uM 5'AZA. RPMI-1640 medium was supplemented with 15% fetal bovine serum, 100 U/ml penicillin, and 100 mg/ml streptomycin.

### Chemicals and antibodies

MG132, 5'-AZA, and cycloheximide (CHX) were purchased from Sigma-Aldrich. Glutathione SepharoseTM 4B was obtained from GE Healthcare. The Ni-NTA Agarose was purchased from Qiagen. ATM inhibitor KU-55933 was purchased from Abcam. Mouse anti-Flag, Myc, HA, and GAPDH antibodies were purchased from Sigma. pS/TQ antibody was from Abcam. Antibodies against β-actin and horseradish peroxidase (HRP)-coupled secondary antibodies were obtained from Santa Cruz Biotechnology (Santa Cruz, USA). Alexa Fluor 488 and 546 were obtained from Life Technologies. The antibiotics and medium were obtained from Invitrogen (Carlsbad, USA).

### Immunofluorescence

Briefly, the indicated cells were fixed with 4% paraformaldehyde in PBS for 10 min. Cells were permeabilized with 0.25% Triton X-100 in PBS for 10 min and stained with indicated antibody at 4°C overnight. Nuclei were stained with DAPI for 1 min. The fluorescence images were acquired using a confocal laser-scanning microscope.

### Western blot and immunoprecipitation

The cells were washed and then lysed in radioimmunoprecipitation assay buffer. The lysate was separated and transferred electrophoretically to PVDF membranes which were blocked and probed with the primary antibody at 4°C overnight. The PVDF membrane was next incubated with the secondary antibody (Bio-Rad) for one hour at room temperature and detected with enhanced chemiluminescence. IP was performed as described previously 11.

### Cell viability assay

Cells (1 × 10^5^/ml) were seeded were seeded in triplicate into 96-well plates. 48 h later, 10 μl of CCK-8 (Beyotime Biotechnology, China) was added to each well. And 4 hours later, the absorbance at 450 nm was measured by a microplate reader (Bio-Rad).

### Flow cytometry

Cells were treated as indicated, harvested, washed with phosphate-buffered saline (PBS; Sigma-Aldrich, P4417). Cell death assay was performed using Annexin V (BD, 556419). Briefly, cells were suspended in Annexin V binding buffer (10 mM HEPES, pH 7.4, 2.5 mM CaCl_2,_ 140 mM NaCl) and stained with Annexin V. Samples were analyzed on the BD LSRII Flow Cytometer (BD Biosciences, USA). FlowJo V7 software (Tree Star Inc., USA) was used to calculate the percentage of cells positive for Annexin V and propidium iodide (PI). Both Annexin V- and PI-negative staining represents viable cells, early apoptotic cells were positive for Annexin V staining, both Annexin V and PI-positive staining means late apoptosis, but necrotic cells were positive for PI staining. Cell cycle experiments were analyzed by flow cytometry with PI staining.

### Mass spectrometry

HEK293 cells were transfected with the human Flag-tagged UCK1 plasmid. Mass spectrometry was performed as described previously 11.

### Quantitative real-time PCR

We purified total RNA from cells using the RNeasy kit (Qiagen). And the Omniscript Reverse Transcription kit (Qiagen) was used for reverse-transcription. The primer sequences for qRT-PCR analysis are listed in **Table S1**. For RT-PCR, reactions were processed and analyzed on an ABI 7700 Sequence Detection System (Applied Biosystems).

### *In vitro* ubiquitination

Flag-UCK1 and Myc-KLHL2 were separately transfected into HEK293 cells. 48 h later, Flag-UCK1 and Myc-KLHL2 were purified with antibodies against Flag and Myc, respectively. Then these proteins were added to the reaction mixture containing adenosine triphosphate (ATP), HA-Ub, E1 and E2 (Boston Biochem, MA). The reaction was stopped and IP with Flag antibody and subsequent IB assay were performed to measure co-IP of UCK1.

### Proximity ligation assay

Proximity ligation assay (PLA) was carried out using Duolink^®^ In Situ Red Starter Kit Mouse/Rabbit (Cat#: DUO92101, Sigma) according to the manufacturer's instructions.

### AML cell-derived xenograft mouse experiment

All animal experiments strictly followed an approved Institutional Animal Care and Use Committee protocol. Mice were housed in sterile conditions using micro-isolators and fed with irradiated food containing antibiotics and acidified water. NOD/SCID mice were bought from Shanghai Laboratory Animal Center. Adult NOD/SCID male mice (6-8 weeks old) were sublethally irradiated and then 10 million per 200 μl HL-60 cells with vector or USP28 overexpression were injected intravenously through mouse tail vein, respectively. These 2 groups were further divided into 4 groups, each containing 10-15 mice: HL-60-vector, HL-60-USP28, HL-60-vector (5'-AZA) and HL-60-USP28 (5'-AZA). 6 days later, the mice were administered 5'-AZA (2.5 mg/kg) for 7 consecutive days once per day or untreated as the control. Then weight loss and survival times of the mice were analyzed.

### Luciferase reporter assay

HEK293T cells were co-transfected transiently with firefly luciferase reporter, the renilla luciferase, and other indicated plasmids. 36 hours later, cells were collected in lysis buffer (25 mM dithiothreitol, 25 mM Tris-Cl (pH 7.8), 2 mM 1,2-diaminocyclo-hoxane N,N,N,N′-tetracetic acid, 1% Triton X-100, and 10% glycerol). Then luciferase assays were carried out with the dual-luciferase reporter assay system (Promega).

### Confocal microscopy

HEK293T cells were transfected with KLHL2 and UCK1 plasmids alone or together for 48 hours, then plated on glass coverslips in six-well plates. Cells were then labeled using indicated antibody. Confocal microscopy image capture and analysis were performed on a Nikon A1 and the Nikon Elements software suite.

### GST pull-down assays

The cDNAs encoding KLHL2 or USP28 was cloned into a pGEX-4T-1 vector (GE Healthcare). cDNAs encoding UCK1 were inserted into pET-22b(+) (Novagen). The expressions of GST, 6× His fusion proteins and the GST pull-down assays, were performed as previously described 12.

### Deubiquitination assay *in vitro and in vivo*

Cells stably expressing Control or USP28 shRNAs (shRNA-#1: GGAGTGAGATTGAACAAGA; shRNA-#2: GTATGGACAAGAGCGTTGG) were transfected with Flag-USP28 wild-type (WT) or Mutant Cys 171 to Ala (CA mutant). Then cells were treated with a proteasome inhibitor, MG132, for four hours. And assays were performed as described previously 13.

### Statistical analysis

All statistical analyses were conducted by Student's t-test using Prism (version 5.0; GraphPad). Experiment or assay was performed at least three times, and representative examples are shown. Data presented represent the mean ± standard error except otherwise demonstrated. *P* < 0.05 was considered statistically significant.

## Results

### KLHL2 directly interacts with UCK1 in the cytoplasm

**Figure 1:**
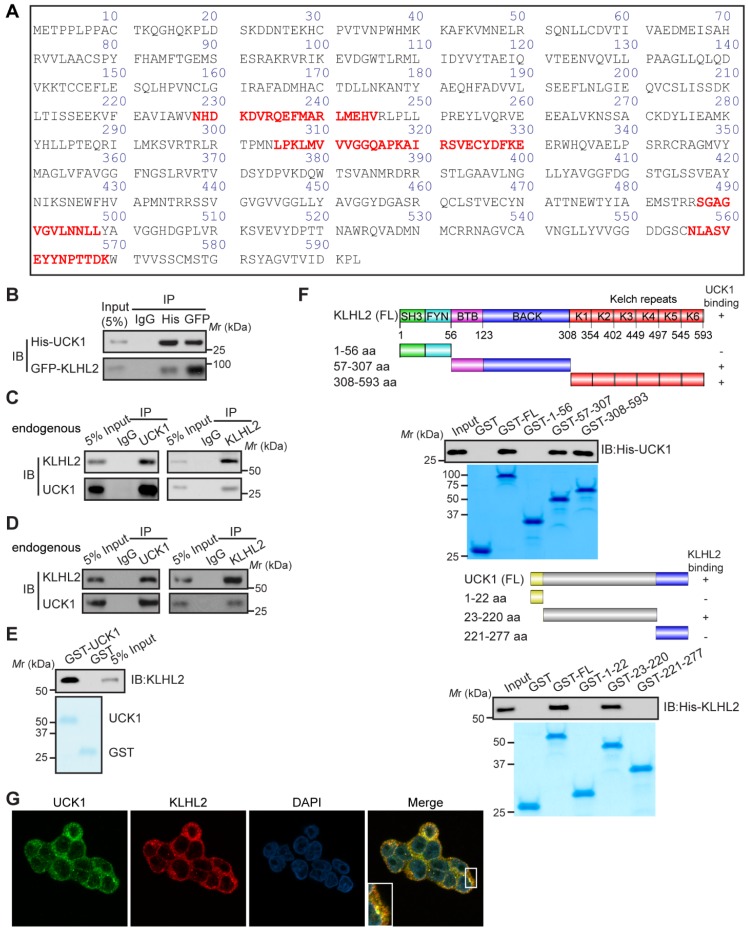
** KLHL2 directly interacts with UCK1 in the cytoplasm.** (**A**) Affinity-purification assay was performed using an anti-Flag-specific antibody and the unique peptides of KLHL2 identified by MS/MS are shown and highlighted in red. (**B**) HEK293T cells were transiently transfected with His-UCK1 and GFP-KLHL2 for 48 hours. Cell lysates were subjected to indicated immunoprecipitation and subsequent immunoblotting with His or GFP antibodies. (**C**) HEK293T cells were extracted and immunoprecipitated with an anti-UCK1 (left panel) or anti-KLHL2 (right panel) antibody and probed with indicated antibodies*.* (**D**) MV4-11 cells were extracted and immunoprecipitated with an anti-UCK1 (left panel) or anti-KLHL2 (right panel) antibody and probed with indicated antibodies*.* (**E**) Recombinant GST-UCK1 protein was incubated with His-tagged KLHL2 protein. Pull-down assay was carried out. (**F**) Binding of different human KLHL2 truncated fragments to UCK1 as indicated (upper panel). Binding of several different domains of human UCK1 to KLHL2 (lower panel). Numbers represent the amino acid (aa) residues in the various domains of KLHL2 and UCK1, respectively. The interaction between UCK1 and KLHL2 domains is indicated by plus sign (+) while minus sign (-) represents no binding between two proteins. Immunoblotting assay of the interaction between indicated constructs with indicated antibodies. Coomassie blue staining were presented to show expression of GST tagged proteins. (**G**) Confocal microscopy of HEK293T cells stained with UCK1 and KLHL2 antibodies. DAPI (blue channel) represents nuclear staining. Red, green and blue channel images were captured by Nikon A1 and the Nikon Elements software suite. Maximum projection images were shown and original magnification ×120.

We sought to identify specific enzymes mediating UCK1 ubiquitination. To this end, we performed a mass spectrometric analysis of Flag-tagged UCK1 in HEK293T cells. KLHL2, one of the ubiquitination-associated enzymes, was identified as an UCK1-interacting protein (**Table S2**), and the unique peptides of KLHL2 identified by mass spectrometry are highlighted in **Figure 1A**. Next, using a reciprocal co-immunoprecipitation (co-IP) assay in cultured cells (**Figure 1B**, **C** and** D**), we demonstrated their physical interaction, which was further confirmed using a GST pull-down assay (**Figure 1E**).

Additionally, both the middle fragments and C terminus of KLHL2 were required for binding to UCK1 (**Figure 1F**, upper panel). And KLHL2 mainly interacted with the 23-220 amino acid (aa) fragment of GST-UCK1, but not with N- or C-terminal fragment of UCK1 (**Figure 1F**, lower panel). To further characterize this interaction, HEK293T cells were transfected with UCK1 and KLHL2. The immunofluorescence confocal microscopic analysis confirmed that UCK1 was mainly co-localized with KLHL2 in the cytoplasm (**Figure 1G**). The above data indicated that KLHL2 directly interacts with UCK1, suggesting the possible involvement of KLHL2 in ubiquitination of UCK1.

### KLHL2 induces K48-linked ubiquitination of UCK1 at K81

**Figure 2:**
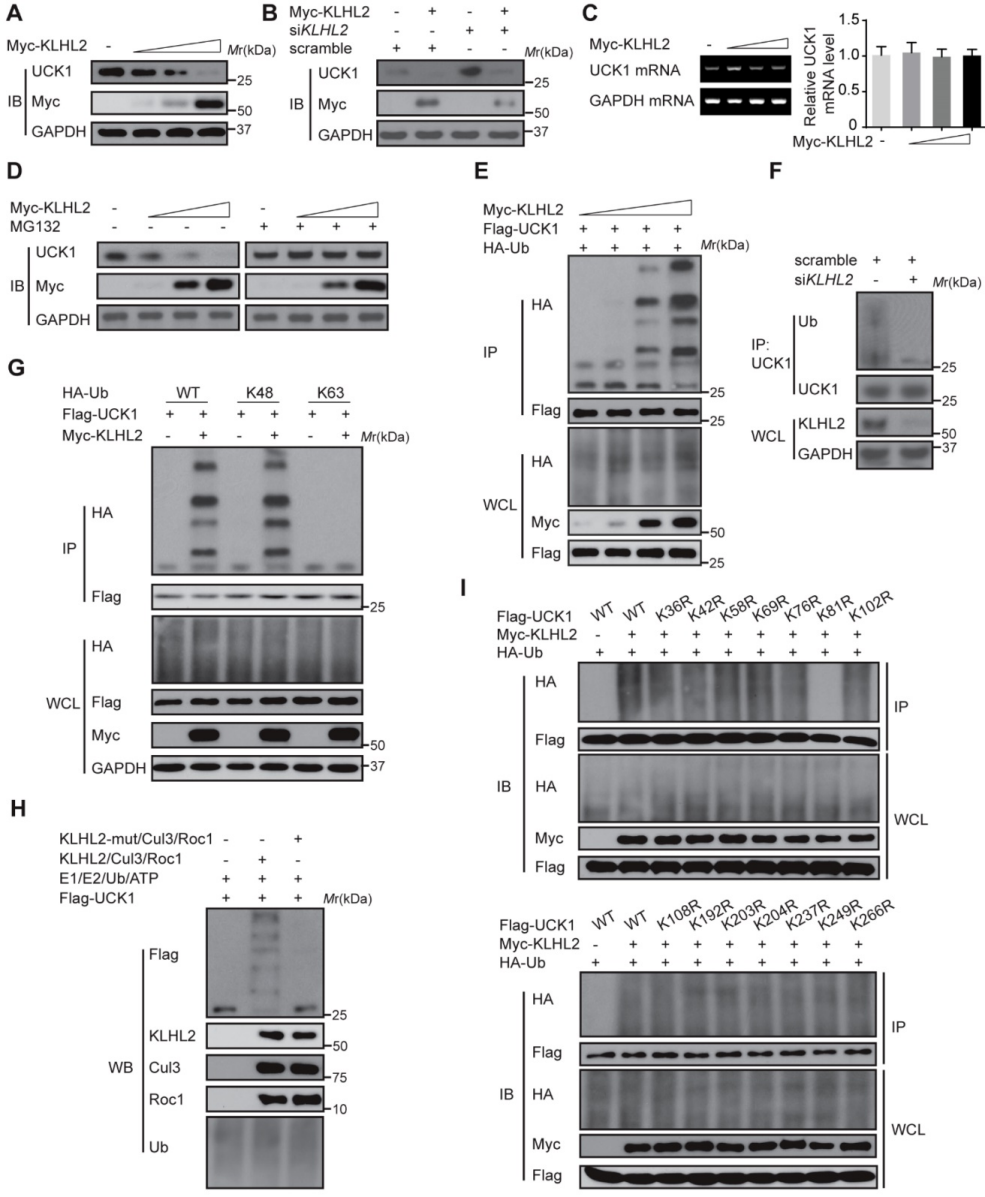
** KLHL2-mediated K48-linked ubiquitination and degradation of UCK1.** (**A**) Immunoblotting assay of UCK1 in HEK293T cells transfected with increasing doses of Myc-KLHL2 plasmids. (**B**) Immunoblotting analysis of HL-60 cells transfected with KLHL2 plasmids together with KLHL2 siRNA as indicated. (**C**) qRT-PCR assay of UCK1 mRNA levels in HEK293T cells transfected with increasing doses of KLHL2 plasmids. GAPDH mRNA serves as a loading control. (**D**) Immunoblotting assay of extracts from HL-60 cells transfected with UCK1 and KLHL2 plasmids and treated with MG132 or dimethyl sulfoxide (DMSO). (**E**) Immunoblotting assay of lysates from HEK293T cells transfected with plasmids for Flag-UCK1, HA-ubiquitin and increasing concentrations of Myc-KLHL2 (0, 1, 1.5 and 2 μg), followed by immunoprecipitation with anti-Flag, and analyzed via Immunoblotting with anti-HA antibody. Cells were treated with MG132 before harvest. (**F**) HL-60 cells were transfected with or without KLHL2 siRNA for 48 h. Then cells were treated with MG132 before harvest. Finally immunoprecipitation were conducted and immunoblotting were shown with indicated antibodies. (**G**) Immunoblotting assay of lysates from HEK293T cells transfected with various combinations of plasmids for Myc-KLHL2, Flag-UCK1, HA-K48-ub and HA-K63-ub and then performed as in **E**. (**H**) *In vitro* ubiquitination assay for UCK1. Flag-UCK1 was subject to ubiquitination in the presence of E1, E2, ATP, and E3 ligase complex. Western blot was performed with antibody as indicated. (**I**) Immunoprecipitation and immunoblotting assays of the indicated proteins in HEK293T cells transfected with KLHL2 and K48-linked ubiquitin together with various UCK1 mutants as shown.

We next examined the effect of KLHL2 on UCK1 expression. As shown in **Figure 2A** and** B**, KLHL2 overexpression significantly decreased the UCK1 protein level, while knockdown of KLHL2 had an opposite effect. Since overexpression of KLHL2 did not change the UCK1 mRNA level (**Figure 2C**), we excluded the possibility that the decrease in the UCK1 protein level resulted from lower expression of the UCK1 gene. The proteasome inhibitor MG132 significantly blocked KLHL2-induced decrease of the UCK1 protein level (**Figure 2D**), showing that KLHL2 reduced UCK1 expression via the proteasome pathway.

Thus, we hypothesized that KLHL2 might ubiquitinate UCK1 and regulate its turnover. To test this hypothesis, KLHL2 and UCK1 were co-transfected in HEK293T cells. Immunoprecipitation and immunoblot analysis showed that KLHL2 could ubiquitinate UCK1 in a dose-dependent manner (**Figure 2E**). Conversely, KLHL2 depletion significantly decreased UCK1 ubiquitination (**Figure 2F**). We also observed that KLHL2 overexpression enhanced K48-linked but not K63-linked ubiquitination of UCK1 (**Figure 2G**). Furthermore, in the presence of Roc1-Cul3-KLHL2 complex 14, UCK1 underwent *in vitro* polyubiquitination, which was abrogated by replacement of KLHL2 with a mutant that could not bind Cul3 (**Figure 2H**). These data indicated that UCK1 is a direct and physiological substrate of KLHL2-based E3 ligase.

We then attempted to identify the specific sites of KLHL2-mediated ubiquitination in UCK1. Among all UCK1 truncations examined, the N- and C-terminal fragments did not show enhanced K48-linked ubiquitination and degradation upon KLHL2 overexpression (**Figure S1**), suggesting that the middle domain (23-220 aa) of UCK1 might contain sites for KLHL2-mediated ubiquitination. Since there are 14 lysine residues in the UCK1 protein, we substituted them with arginine to create 14 single-site mutants. We observed that the K81R mutation almost completely blocked KLHL2-induced ubiquitination of UCK1 (**Figure 2I** and **Figure S2**), suggesting that K81 is required for KLHL2-mediated UCK1 degradation.

### Silencing KLHL2 chemosensitizes AML cells to 5'-AZA

**Figure 3:**
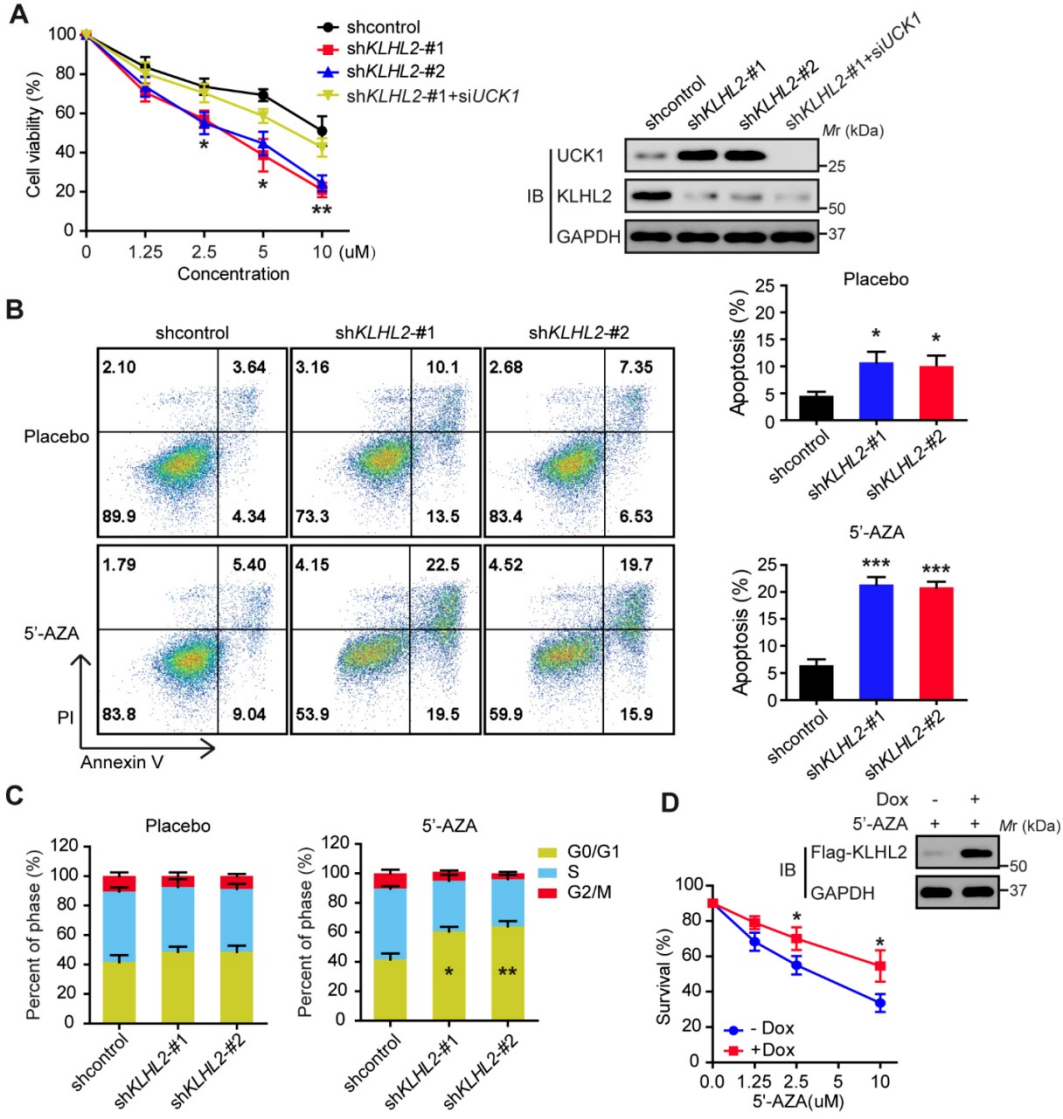
** Knockdown of KLHL2 significantly sensitizes 5'-AZA to inhibit AML cells.** MV4-11 cells transfected with *KLHL2* shRNA and shcontrol along with UCK1 siRNA or without were treated with or without 5'-AZA for 48 h. (**A**) CCK8 assays were carried out to examine the proliferation of MV4-11 cells in the presence of different concentrations of 5'-AZA (left panel). The protein levels of UCK1 and KLHL2 were determined (right panel) (**B**) FACS analysis of the apoptosis of MV4-11 cells under 2.5 uM 5'-AZA. Representative images of cellular apoptosis were shown (left panel). And the percentage of apoptosis cells with indicated treatment was presented in the bar charts (right panel). (**C**) The percentage of cells with indicated treatment in the different phases of the cell cycle was shown in the bar charts. (**D**) MV4-11 cells were transfected with a doxycycline-inducible lentiviral vector to upregulating Flag-KLHL2. 24 h later, cells were treated with increasing doses of 5'-AZA. Then cell survival was determined using Annexin V staining (left panel). Cell lysates were analyzed by Western blots using anti-Flag antibody to confirm doxycycline-inducible expression (right panel). *P < .05; **P < .01, and ***P < .001.

Since UCK1 is a key player in the 5'-AZA metabolism and activation 4, we next investigated the chemosensitizing effects of KLHL2 inhibition on AML cell survival. To this end, *KLHL2*-silenced and scramble MV4-11 cells were cultured in the presence of 5'-AZA. The CCK8 assay indicated that compared with the shcontrol cells, the MV4-11 cells with *KLHL2* knockdown were more prone to inhibition, while the simultaneous loss of UCK1 abolished this inhibition (**Figure 3A**), suggesting that UCK1 loss mediates the effect of KLHL2 on 5'-AZA treatment in AML cells. Consistent with this observation, silencing *KLHL2* significantly enhanced the effect of 5'-AZA on the apoptosis of MV4-11 cells (**Figure 3B**). Moreover, *KLHL2* knockdown combined with 5'-AZA treatment resulted in a significantly higher number of MV4-11 cells arrested in the G0/G1 phase (**Figure 3C**), indicating a synergistic interplay between 5'-AZA treatment and *KLHL2* silencing. A similar phenotype was observed in other AML cell lines (**Figure S3**).

To further validate these findings, a doxycycline-inducible lentiviral system was used to manipulate KLHL2 expression in MV4-11 cells. We found that doxycycline-induced KLHL2 upregulation significantly contributed to enhanced chemotherapeutic resistance to 5'-AZA (**Figure 3D** and**Figure S3D**). Therefore, targeting KLHL2 sensitizes AML cells to 5'-AZA chemotherapy.

### USP28 antagonizes KLHL2-mediated UCK1 ubiquitination and degradation

**Figure 4:**
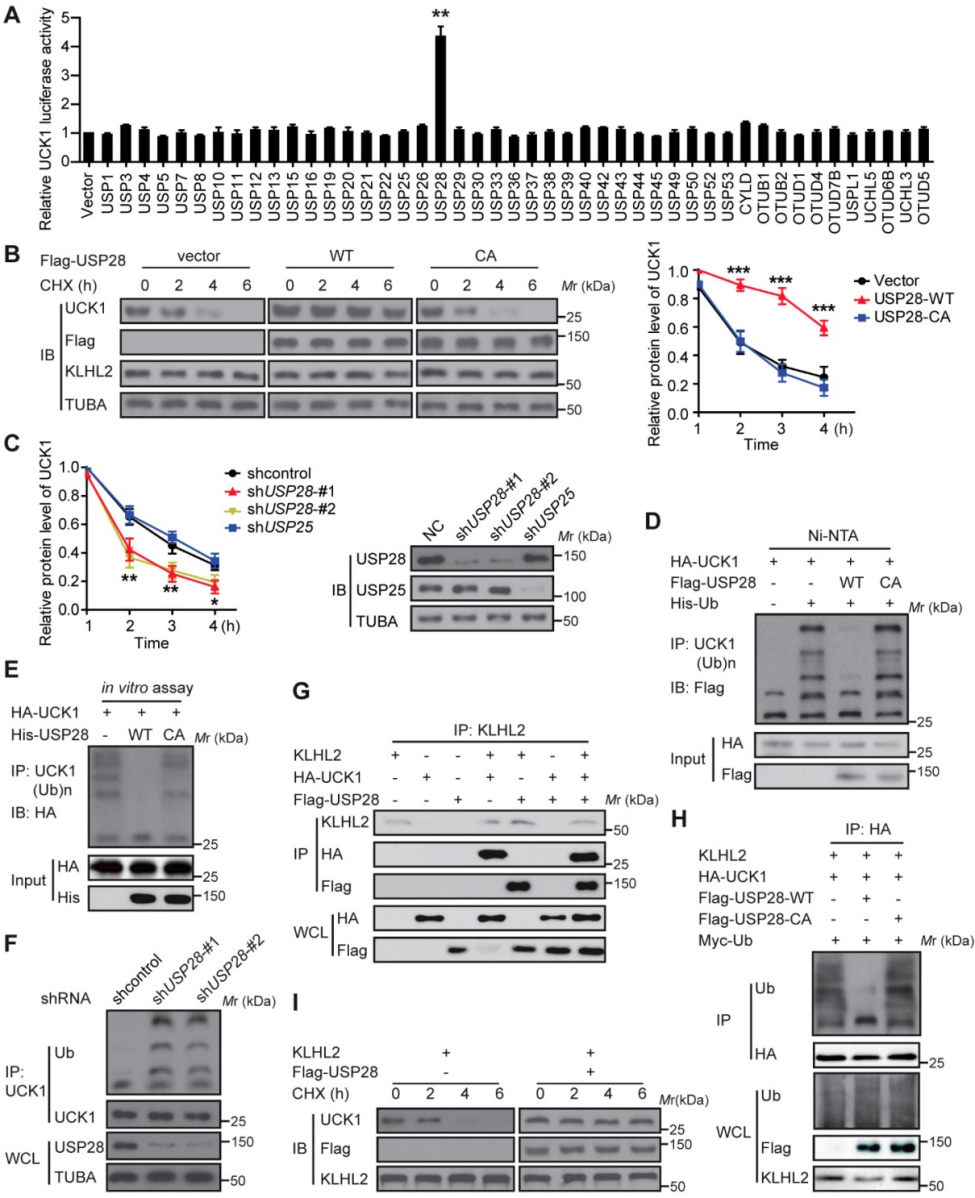
** USP28 directly counteracts KLHL2-mediated UCK1 ubiquitination and proteasomal degradation.** (**A**) Screening the deubiquitinating enzymes of UCK1. The constructs as indicated were transfected into HEK293T cells. Then the UCK1-Luc firefly luciferase activity was measured. (**B**) In HL-60 cells, Flag vector, Flag-USP28-WT or Flag-USP28-CA (C171A), were expressed and treated with CHX (50 μg/ ml) for indicated time intervals. Then the protein levels were analyzed by western blotting (left panel) and statistical analysis of UCK1 was presented (right panel). (**C**) MV4-11 cells were transfected with control shRNA, USP28 shRNA-#1, USP28 shRNA-#2, or USP25 shRNA, and then treated with CHX. Western blotting was performed and statistical analysis of UCK1 level was presented (left panel). The graph represents mean ± SD. Western blots were also used to examine the protein levels of USP28 and USP25 (right panel). (**D**) HA-UCK1 and His-ubiquitin were co-expressed with WT-USP28 or catalytic-dead USP28 (C171A) in HEK293T cells. After MG132 (10 μM) treatment for 6 h, Ni-NTA agarose beads were used to pull down the ubiquitinated proteins under denaturing conditions, and the ubiquitination of UCK1 was detected by western blotting. (**E**) His-USP28 protein and HA-UCK1 were subjected to *in vitro* deubiquitination. (**F**) MV4-11 cells stably expressing control or USP28 shRNA-#1, shRNA-#2, were treated with MG132 for 6 h. UCK1 was immunoprecipitated with an anti-UCK1 antibody. Immunoblotting was performed with indicated antibodies. (**G**) KLHL2 binds to USP28. HEK293T cells were transfected with indicated plasmids. Cell lysates were subjected to immunoprecipitation with anti-KLHL2 antibody and immunoblotting analysis. (**H**) USP28 inhibited KLHL2-mediated UCK1 ubiquitination. HEK293T cells were transfected with indicated plasmids as indicated and immunoblotting was carried out. (**I**) KLHL2-mediated proteasomal degradation of UCK1 was suppressed by USP28. HL-60 cells were transfected with indicated plasmids. 24 hours later, cells were cultured in the presence of CHX for the indicated time. Then immunoblotting analysis was performed.

We subsequently searched for DUBs that could antagonize KLHL2 and stabilize UCK1. To this end, a fusion protein (UCK1-Luc) was constructed by fusing firefly luciferase to the C-terminus of UCK1 and used as a reporter of UCK1 stability. Since the fused luciferase was degraded together with UCK1, the degradation of UCK1 could be observed by checking the luciferase activity. We found that wild-type (WT)-USP28, but not the other 45 mammalian DUBs, not only significantly increased the luciferase activity of UCK-Luc (**Figure 4A**), but also prolonged the half-life of UCK1 (**Figure 4B**). Because the catalytic-inactive mutant C171A (USP28-CA) did not stabilize UCK1, the stabilization of UCK1 by USP28 was dependent on its deubiquitinating enzyme activity (**Figure 4B**). Conversely, depletion of endogenous USP28 by shRNA in MV4-11 cells reduced the half-life of UCK1 (**Figure 4C**). Although USP25 is the most closely related DUB to USP28 15, silencing USP25 in MV4-11 cells had little effect on the half-life of UCK1 (**Figure 4C**), suggesting that USP28 might be a specific DUB that regulates UCK1 stability.

We then evaluated whether USP28 could deubiquitinate UCK1. Our data showed that WT-USP28, but not the USP28-CA, almost entirely removed the ubiquitin chains from UCK1 (**Figure 4D**). *In vitro* deubiquitination assay further confirmed that WT-USP28, but not USP28-CA, efficiently deubiquitinated UCK1 (**Figure 4E**), indicating that USP28 served as direct DUB for UCK1. Conversely, USP28 silencing in HEK293T markedly increased the ubiquitination of endogenous UCK1 (**Figure 4F**), implying that endogenous UCK1 was also a target of USP28.

Given the above findings, we hypothesized that USP28 might restrain UCK1 ubiquitination induced by KLHL2. To test this hypothesis, we first expressed KLHL2, HA-UCK1, and Flag-USP28 in HEK293T cells and investigated the interaction between these proteins. IP and IB analyses suggested interactions between KLHL2, UCK1, and USP28 (**Figure 4G**). We next examined the effect of USP28 on KLHL2-mediated ubiquitination of UCK1. Intracellular ubiquitination assays indicated that WT-USP28 decreased the level of KLHL2-mediated ubiquitination of UCK1, while USP28-CA failed to dramatically restrain UCK1 ubiquitination mediated by KLHL2 (**Figure 4H**). These data were further supported by the observation that USP28 significantly rescued elevated instability of UCK1 protein in the presence of KLHL2 (**Figure 4I**). We also confirmed that the USP28 knockdown did not regulate the protein level of KLHL2 (**Figure S4B**). Together, these findings validate the opposite role for KLHL2 and USP28 in the ubiquitination and turnover of UCK1.

### USP28 binds to UCK1 via KLHL2

Although KLHL2 and UCK1 formed binary complexes, USP28 did not directly bind to UCK1. Given that several DUBs are recruited to their substrates indirectly through binding to the E3 ligase 16, we analyzed whether KLHL2 mediated the interaction between UCK1 and USP28 *in vivo*. Consistent with this notion, no complex formation was observed upon co-transfection of HA-USP28 and UCK1 in HEK293T cells, but coexpression of KLHL2 strongly stimulated the interaction between HA-USP28 and UCK1 (**Figure S5A**). Conversely, silencing KLHL2 significantly decreased the amount of endogenous UCK1 that was bound to HA-USP28 (**Figure S5B**). Our data strongly suggest that KLHL2 mediates the interaction between UCK1 and USP28.

### ATM-mediated phosphorylation of USP28 and UCK1 has an opposite effect on their association with KLHL2

**Figure 5:**
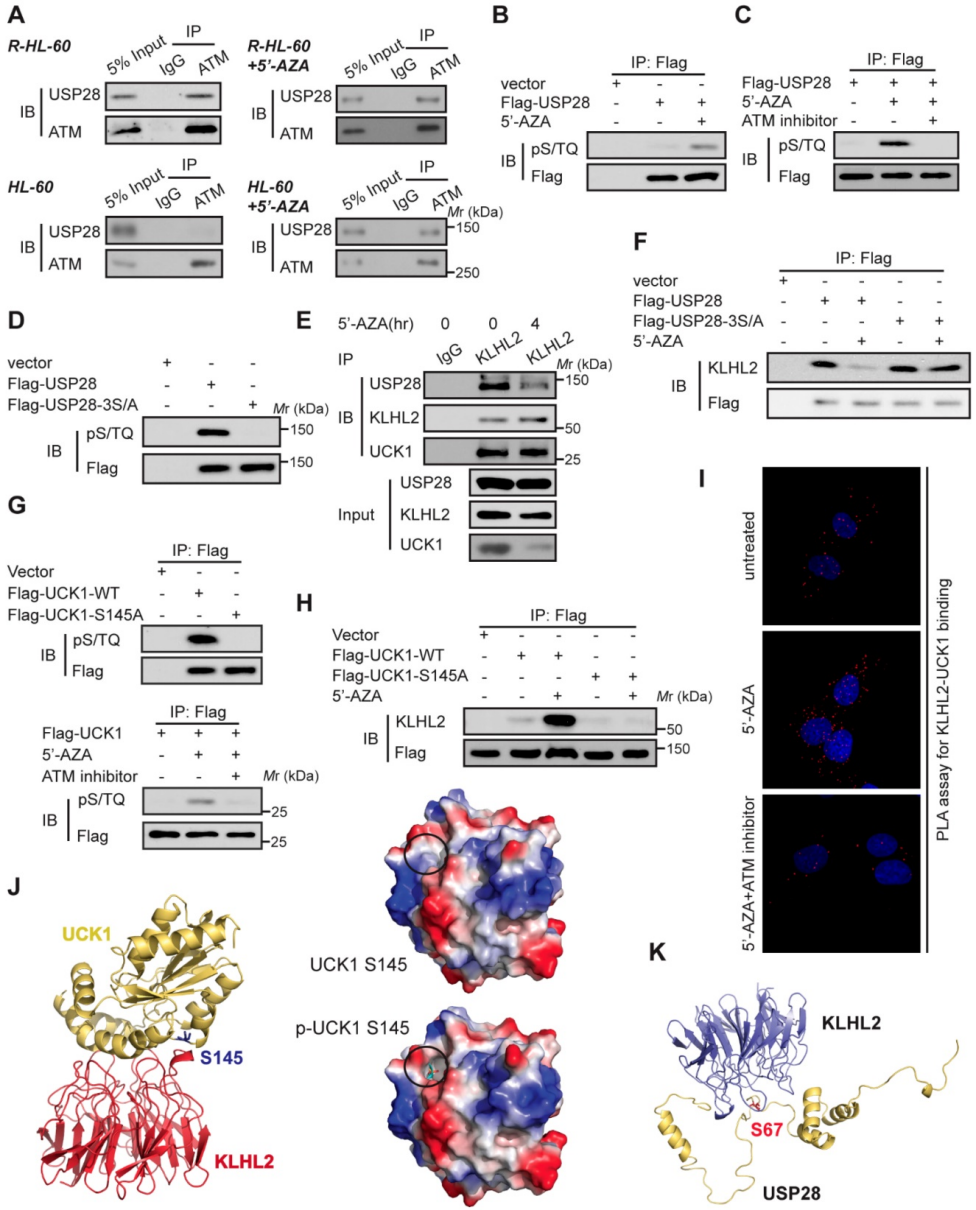
** ATM-mediated phosphorylation of USP28 and UCK1 and its physiological significance.** (**A**) ATM interacted with USP28. Cell lysates were collected from 5'-AZA resistant R-HL-60 cells with or without 5'-AZA treatment (upper panel), or HL-60 cells with or without 5'-AZA treatment (lower panel) respectively. Then immunoprecipitation and immunoblotting were performed. (**B and C**) ATM phosphorylated USP28 in response to 5'-AZA treatment. HL-60 cells expressing Flag-USP28 were treated with or without 5'-AZA for 6 h (**B**), or pre-treated with the ATM inhibitor KU-55933 for 1 h before 5'-AZA treatment (**C**), cell lysates were collected, and immunoprecipitation followed by immunoblotting. (**D**) USP28 was phosphorylated on its S/TQ motifs. HL-60 cells were transfected with vector, Flag-USP28-WT or Flag-USP28-3S/A, in which all the three S/TQ sites within the USP28 polypeptide were mutated to A (USP28-3S/A). 48 h later, immunoblotting was performed. (**E**) The interaction between KLHL2 and USP28 significantly decreased under 5'-AZA treatment. Cell lysates were collected from HL-60 cells with or without 5'-AZA treatment for 4 h and immunoprecipitated with an anti-KLHL2 antibody. (**F**) Phosphorylation-deficient USP28-3S/A stably bind to KLHL2 after 5'-AZA treatment. HL-60 cells expressing Flag-USP28 or Flag-USP28-3S/A were treated with 5'-AZA for 4 h, cell lysates were harvested. (**G**) UCK1 was phosphorylated by ATM in response to 5'-AZA treatment. HL-60 cells expressing Flag-UCK1-WT or Flag-UCK1-S145A were treated with or without 5'-AZA for 6 h (upper panel), or pre-treated with KU-55933 for 1 h before 5'-AZA treatment (lower panel), cells were harvested and subjected to immunoprecipitation followed by immunoblotting. (**H**) UCK1 was phosphorylated on its S/TQ motifs. HL-60 cells were transfected with vector, Flag-UCK1-WT or Flag-UCK1-S145A. 2 days later, these cells were treated with or without 5'-AZA for 6 h, then lysates were subjected to immunoprecipitation followed by immunoblotting. (**I**) PLA of the interaction between UCK1 and KLHL2 in HL-60 cells with indicated treatment. (**J**) Based on the prediction in ZDOCK and pymol software, Ser145 of UCK1 lays in the interface between KLHL2 and UCK1, and Ser145 phosphorylation greatly enhances the binding affinity of UCK1 to KLHL2 by increasing hydrophilicity. (**K**) Structural docking modeling predicted in ZDOCK and pymol software reveals that Ser67 of USP28 lays in the interface between KLHL2 and USP28.

Since 5'-AZA treatment can activate ATM in AML cells and the ATM pathway can prevent the apoptosis induction in 5'-AZA resistant cells 17, we sought to determine whether ATM modulated the effect of USP28 on the UCK1 deubiquitination in response to 5'-AZA. First, Co-IP assays revealed the association of USP28 with ATM in 5'-AZA resistant R-HL-60 cells (**Figure 5A,** upper panel), which was not observed in HL-60 cells without 5'-AZA resistance (**Figure 5A**, lower panel). Second, we found that the ATM substrate pS/TQ antibody was reactive with the immunoprecipitated Flag-USP28 and 5'-AZA treatment significantly increased this reactivity in HL-60 cells (**Figure 5B**). However, pretreatment of HL-60 cells by KU-55933, an ATM-specific inhibitor 18, markedly diminished this reactivity (**Figure 5C**). Once all the three conservative SQ sites (S67, S495, and S714), in USP28, were mutated to alanine, the pS/TQ antibody completely could not recognize the immunoprecipitated Flag-USP28-3S/A (**Figure 5D**), suggesting that in response to 5'-AZA-induced stress, ATM phosphorylates USP28 at all these three sites.

We then examined the biological significance of ATM-mediated phosphorylation of USP28. We found that upon 5'-AZA treatment, the amount of USP28 in the KLHL2 immunocomplex significantly decreased (**Figure 5E**), whereas the interaction between USP28-3S/A and KLHL2 did not change (**Figure 5F**). These findings suggested that ATM-mediated phosphorylation of USP28 promotes dissociation of USP28 from KLHL2, destabilizing UCK1.

Interestingly, we also found that ATM phosphorylated UCK1 at S145 but not at S63 (**Figure 5G**), and UCK1 phosphorylation at S145 significantly enhanced the KLHL2-UCK1 complex formation (**Figure 5H**). Proximity ligation assay (PLA) further confirmed the effect of UCK1 phosphorylation at S145 by ATM on the interaction between UCK1 and KLHL2 (**Figure 5I**). Consistent with our data, computational modeling of structures predicted by the ZDOCK and PyMol software showed that Ser145 is the critical amino acid mediating the direct binding of UCK1 to KLHL2 (**Figure 5J**) while Ser67 phosphorylation disrupts the interaction between USP28 and KLHL2 (**Figure 5K**).

### Ectopic USP28 expression significantly enhances the inhibitory effects of 5'-AZA on AML cells *in vitro* and *in vivo*

**Figure 6:**
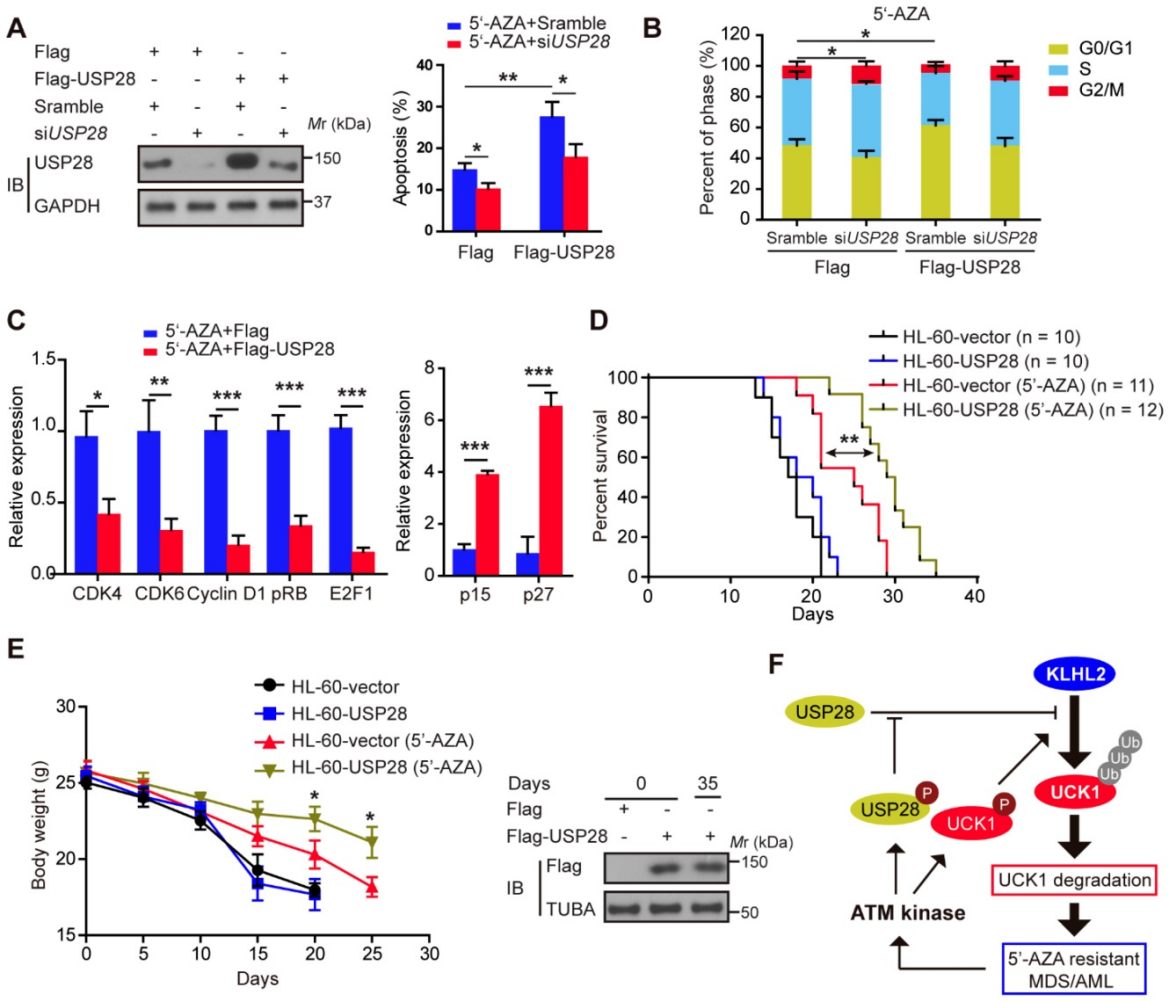
** Ectopic USP28 expression significantly sensitized 5'-AZA to inhibit HL-60 cells *in vitro* and *in vivo*.** HL-60 cells were divided into HL-60-vector and HL-60-USP28. (**A**) The cells were treated with 5'-AZA or 5'-AZA plus *USP28* siRNA. 24 hours later, the apoptosis was analyzed by flow cytometry (right panel). Western blots were performed to examine the level of USP28 (left panel). (**B**) Effects of 5'-AZA on cell cycle progression of HL-60 after USP28 silencing or upregulating. (**C**) Effects of USP28 overexpression on 5'-AZA-regulated cell cycle-related genes in HL-60 cells which were quantified by qRT-PCR. (**D**) Kaplan-Meier survival curves for recipients of HL-60 cells with or without USP28 transfection. (**E**) Left panel: Body weight for AML mice with indicated treatment. Results were expressed as mean ± SEM of at least 10 mice in each group. Right panel: protein samples were extracted from HL-60 cells (the first two lanes) or mouse spleen (the last lane) and then western blot was performed with antibody as indicated. *P < .05; **P < .01 (**F**) Schematic model indicates that KLHL2/USP28/ATM axis controls UCK1 downregulation which contributes to chemoresistance to 5'-AZA in patients with AML.

We subsequently examined the effect of USP28 on the apoptosis of HL-60 cells after 5'-AZA treatment. Compared with the control groups, the apoptotic rate of the HL-60 cells with USP28 overexpression significantly increased upon 5'-AZA treatment, while silencing USP28 reverted this effect (**Figure 6A**). Moreover, 5'-AZA-induced HL-60 cell apoptosis was accompanied by G0/G1 arrest, especially when USP28 was upregulated (**Figure 6B**). Conversely, USP28 silencing accelerated the cell cycle progression to G2/M phase (**Figure 6B**). As expected, USP28 overexpression further decreased the relative expression of cell cycle-related genes (CDK4, CDK6, Cyclin D1, pRB, and E2F1) induced by 5'-AZA, whereas p15 and p27 expression was significantly enhanced (**Figure 6C**).

We established the *in vivo* intravenous AML mouse model by injecting HL-60 cells transfected with or without USP28. This HL-60 xenotransplanted mouse model kills mice with a short latency like that as in AML patients, causing rapid AML progression in bone marrow, spleen, and liver. Treatment of the AML mice with 5'-AZA significantly inhibited HL-60 cell growth, in particular of the HL-60 cells with USP28 upregulation (**Figure 6D**), and prolonged the overall survival (**Figure 6E**). Similar results were also observed when KLHL2 was knocked down in MV4-11 cells (**Figure S6**), and were further validated in primary AML cells (data not shown). Therefore, USP28 may be a critical target that sensitizes 5'-AZA to treat high-risk AML in clinical practice.

## Discussion

Herein, we examined the involvement of KLHL2 and USP28 in the therapeutic effects of 5'-AZA on AML by modifying the UCK1 protein. We have demonstrated that KLHL2-mediated ubiquitination of UCK1 inhibits the therapeutic effects of 5'-AZA on AML cells. The findings that support this conclusion are the following: (i) the binding of UCK1 to the E3 ligase KLHL2 causes its polyubiquitination at K81 and degradation; (ii) the DUB USP28 binds to UCK1 through interaction with KLHL2 and antagonizes KLHL2-mediated effect on UCK1; (iii) UCK1 deubiquitination by USP28 is dependent on the phosphorylation status of USP28; (iv) USP28 is phosphorylated by 5'-AZA-activated ATM, resulting in disassociation of KLHL2 from USP28 and UCK1 destabilization; (v) ATM also phosphorylates UCK1 at S145, significantly enhancing the KLHL2-UCK1 complex formation; (vi) the knockdown of KLHL2 not only significantly inhibits AML cell proliferation, but also sensitizes AML cells to 5'-AZA-induced apoptosis; and (vii) combinational therapies with 5'-AZA and ectopic USP28 expression enhances the effects of 5'-AZA and induces synergistic lethality at cellular level and in the AML mouse model, which is, however, observed in cells deficient in USP28. These novel findings establish a direct connection between the ubiquitination of UCK1 by KLHL2 and phosphorylation of USP28 and UCK1 by ATM, which are involved in mediating drug resistance against 5'-AZA in AML patients. Therefore, targeting UCK1 ubiquitination and phosphorylation mediated by the KLHL2/USP28/ATM axis may enhance the therapeutic effects of 5'-AZA on AML.

Little is known about the role of KLHL2 in hematological malignancies. KLHL2, also termed Mayven, is a member of the kelch-related superfamily of proteins and predominantly expressed in brain 19. A previous study indicated that overexpression of KLHL2 promotes breast cancer growth 20. In breast cancer, KLHL2 functions as an oncogene by activating c-Jun N-terminal kinase and inducing cyclin D1 expression via its BTB/POZ domain 20. Consistent with these data, we report here that KLHL2 expression contributes to 5'-AZA chemoresistance in AML cells. However, one very crucial finding in our study is that KLHL2 inhibits AML cell apoptosis and cell cycle arrest through its E3 ligase mediated-UCK1 ubiquitination. Given that KLHL2-Cullin3 also increase the ubiquitination of WNK4, which is so far the only known substrate for the KLHL2-Cullin3 E3 ligase complex 21, it is tempting to speculate that KLHL2 might ubiquitinate other unrecognized metabolism-related kinases. Also, although KLHL2 shares the highest similarity with KLHL3 22, we did not observe regulation of UCK1 by KLHL3. Therefore, a better definition of key enzymes in clinical response to 5'-AZA in patients with AML would be helpful in clarifying their specific mechanisms of action and further optimize therapy.

Another exciting finding in this report is that the crosstalk of phosphorylation and ubiquitination dictates the fate of UCK1. Phosphorylation and ubiquitination processes are highly interdependent, where often phosphorylation of a protein decides its ubiquitination 23-26. In response to 5'-AZA, UCK1 and USP28 are phosphorylated by ATM, instigating entirely different effects on the protein-protein interaction. This difference might result from the change in protein structure mediated by phosphorylation. To address this issue, our laboratory is generating antibodies against the UCK1-S145 and USP28-3S phosphorylation sites. It is anticipated that these specific antibodies would not only provide further evidence to support the importance of phosphorylation of UCK1 and USP28 in UCK1 degradation mediated by KLHL2, but also help in screening 5'-AZA chemoresistance in clinical practice.

Consistent with our data as described above, many ubiquitin modifying enzymes including deubiquitinating enzymes have been shown to play important roles in neoplastic diseases 8, 27. Our data suggest that a lower level of USP28 could be used as a biomarker for patients' resistance to 5'-AZA therapy. Also, a recent study reported the importance of USP28 in resistance to other anti-cancer therapies, including BRAF inhibitor therapy in melanoma 28. The loss of USP28 promoted MAPK activation and resistance to RAF inhibitor therapy by stabilizing BRAF in cell culture and *in vivo* models. Importantly, the study indicated that in a proportion of melanoma patients, USP28 was deleted, thereby functioning as a key biomarker for patients' response to the BRAF inhibitor therapy 28. However, USP28 has also been shown to directly stabilize ChK1, FBW7, and FBW7-substrates (c-MYC, HIF-1α, and cyclin E1) 16, 28-34. These findings highlighted a context-dependent oncogenic role of USP28 and led to the identification of several USP28 inhibitors 35.

The identification of the KLHL2/USP28/ATM axis in coordinating UCK1 ubiquitination offers a unique opportunity to counter the chemoresistance to 5'-AZA. Indeed, we observed that KLHL2 knockdown or USP28 overexpression enhances the effect of 5'-AZA at a cellular level and in the AML mouse model. Our findings were supported by a study, which reported that targeting the ATM kinase activities exhibits antitumoral activity in AML 36. Given that 5'-AZA is widely used in the treatment of various human cancers 37, our findings may be relevant for other cancers beyond AML, such as glioblastoma (data not shown), and therefore warrant further investigation.

Although our study has identified KLHL2-mediated ubiquitination as a primary mechanism for UCK1 downregulation, we cannot rule out the involvement of Ubiquitin Proteasome System (UPS) -independent pathway in UCK1 degradation and the 5'-AZA-resistant phenotype. For example, selective autophagy may be another mechanism to degrade UCK1, and requires further investigation. Nevertheless, our study strongly supports that KLHL2-mediated UCK1 downregulation serves as an adaptive mechanism to suppress 5'-AZA-induced AML cell apoptosis.

In summary, the current study identifies the KLHL2/USP28/ATM axis as a master regulator of the 5'-AZA-resistant mechanism by coordinating UCK1 degradation, and suggests that lower activity of USP28 or high expression of KLHL2 might be two important patient stratification biomarkers for the most appropriate use of 5'-AZA. Our data also elucidate potential mechanisms to overcome UCK1-related resistance to 5'-AZA.

## Supplementary Material

Supplementary figures and tables.

## Abbreviations

5'-AZA: 5'-azacytidine
AML: acute myeloid leukemia
UCK1: uridine-cytidine kinase 1
DUB: deubiquitinase
CHX: cycloheximide
PI: propidium iodide
PLA: Proximity ligation assay
KLHL2: Kelch Like Family Member 2
USP28: Ubiquitin Specific Peptidase 28
Dox: Doxycycline
IP: Immunoprecipitation
ATM: Ataxia Telangiectasia Mutated
CDK4: cyclin dependent kinase 4
CDK6: cyclin dependent kinase 6
E2F1: E2F transcription factor 1
P15: cyclin dependent kinase inhibitor 2B
P27: cyclin dependent kinase inhibitor 1B
qRT-PCR: Quantitative real-time PCR
siRNA: Short interfering RNA
