# Supplementary material for: Deubiquitinase USP28 inhibits ubiquitin ligase KLHL2-mediated uridine-cytidine kinase 1 degradation and confers sensitivity to 5'-azacytidine-resistant human leukemia cells
Source: Theranostics. 2020 Jan 1;10(3):1046–59. doi: 10.7150/thno.36503 (PMC6956814; doi:10.7150/thno.36503)
Supplement: Supplementary file 1 — Supplementary figures and tables. [file thnov10p1046s1.pdf]

Deubiquitinase USP28 inhibits ubiquitin ligase KLHL2-mediated uridine-cytidine kinase 1 degradation and confers sensitivity to 5'-azacytidine-resistant human leukemia cells

Heng Zhang[1], He Huang[1, *], Xing Feng[2], Huiwen Song[3], Zhiyong Zhang[4, *], Aizong Shen[5, *], Xingfeng Qiu[6, *]

Supplementary information

Table S1

Primers for qRT-PCR

Gene	Sense primer	Antisense primer
USP28	GGAACAGCAGCAAGATGTGA	GGCCGAAGGTCTCATTGTTA
CDK4	ATGGCTGCCACTCGATATGAACCC	GTACCAGAGCGTAACCACCACAGG
CDK6	TGGAGACCTTCGAGCACC	CACTCCAGGCTCTGGAACTT
RB	GGACATGTGAAC TTATATA	GAACGATTATCCATT CAAA
E2F1	GCCACTGACTCTGCCACCATAG	CTGCCCATCCGGGACAAC
cyclin D1	AAGGCGGAGGAGACCTGCGCG	ATCGTGCGGCATTGCGGC
β-actin	AGCCATGTACGTTGCTAT	GATGTCCACGTCACACTTCA
KLHL2	TTCTTAACCTCGGCATC	AACTCCTGTCTTACGTCCTT
p27	AATAAGGAAGCGACCTGCAA	CCTCCCTTCCCCAAAGTTTA
p15	CGTTAAGTTTACGGCCAACG	GGTGAGAGTGGCAGGGTCT
GAPDH	GCCAAGGTCATCCATGACAAC	GTCCACCACCCTGTTGCTGTA

Table S2

Identification of UCK1-interacting proteins via mass spectrometry

Swiss-Prot accession no.	Gene Symbol	protein name	No. of unique peptides matched	% sequence identified by ms/ms data	Protein mascot score
P00558	PGK1	Phosphoglycerate Kinase 1	7	23%	195
Q9HA47	UCK1	Uridine-Cytidine Kinase 1	8	25%	182
O95198	KLHL2	Kelch Like Family Member 2	4	18%	154
Q9BZX2	UCK2	Uridine-Cytidine Kinase 2	6	20%	128
Q9BZM1	PLA2G12A	Phospholipase A2 Group XIIA	7	17%	120
Q96RU2	USP28	Ubiquitin Specific Peptidase 28	6	13%	107
Q5T6WS	HNRNPK	Heterogeneous Nuclear Ribonucleoprotein K	5	15%	106
Q13315	ATM	Ataxia Telangiectasia Mutated	4	13%	82
K1UQX6	HSP90	Heat shock protein 90	7	17%	63
O14744	PRMT5	Protein Arginine N-Methyltransferase 5	6	16%	42
Q6NYC1	JMJD6	Bifunctional Arginine Demethylase And Lysyl-Hydroxylase JMJD6	8	10%	38

Figure S1

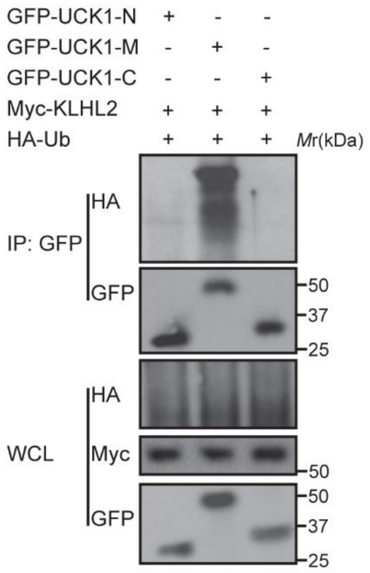

Figure S1. Overexpression of KLHL2 had no effect on the ubiquitination level of N or C terminal region of UCK1. Immunoblotting assay of lysates from HEK293T cells transfected with plasmids for GFP-UCK1-N (N-terminal 1-22 aa), GFP-UCK1-C (C-terminal 221-277 aa), GFP-UCK1-M (middle domain 23-220 aa), HA-ubiquitin and Myc-KLHL2, followed by immunoprecipitation with anti-GFP, and analyzed via Immunoblotting with anti-HA antibody. Cells were treated with MG132 before harvest.

Figure S2

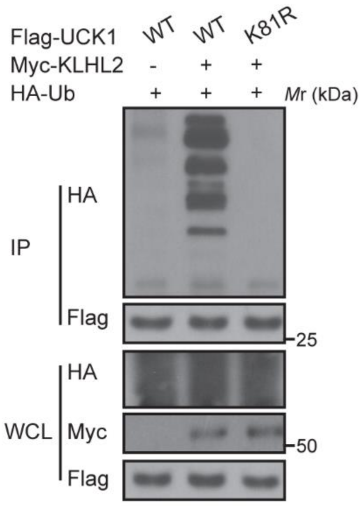

Figure S2. KLHL2 does not degrade UCK1 K81R mutant. Immunoprecipitation and immunoblotting assays of the indicated proteins in HEK293T cells transfected with KLHL2 and K48-linked ubiquitin together with various UCK1 WT or K81R mutant as shown.

Figure S3

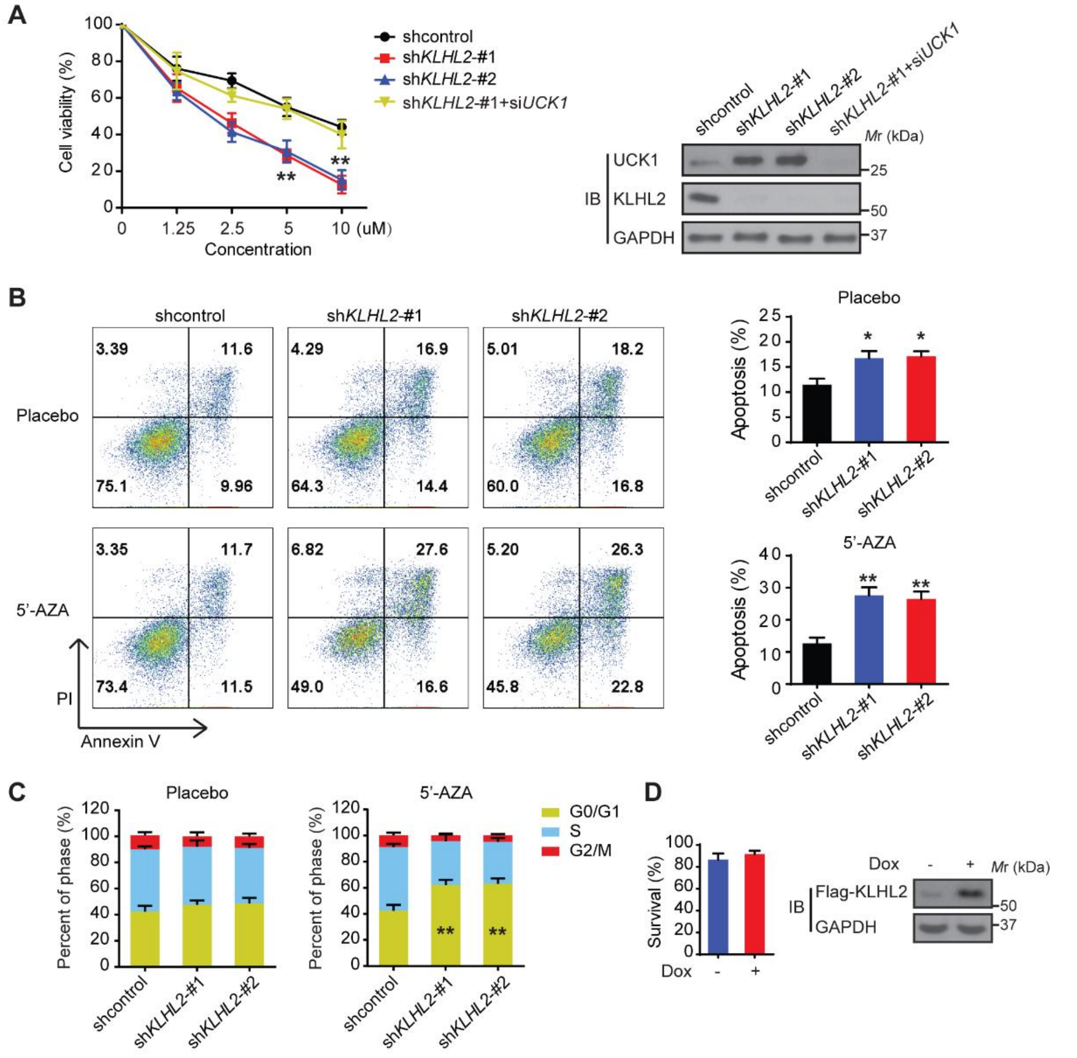

Figure S3. Knockdown of KLHL2 significantly sensitizes 5'-AZA to inhibit AML cells. R-HL-60 cells transfected with *KLHL2* shRNA and shcontrol were treated with or without 5'-AZA for 48 h. (**A**) CCK8 assays were carried out to examine the proliferation of R-HL-60 cells in the presence of different concentrations of 5'-AZA (left panel). The protein levels of UCK1 and KLHL2 were determined (right panel) (**B**) FACS analysis of the apoptosis of R-HL-60 cells under 2.5 uM 5'-AZA. Representative images of cellular apoptosis were shown (left panel). And the percentage of apoptosis cells with indicated treatment was presented in the bar charts (right panel). (**C**) The percentage of cells with indicated treatment in the different phases of the cell cycle was shown in the bar charts. (**D**) MV4-11 cells were transfected with a doxycycline-inducible lentiviral vector to upregulating Flag-KLHL2. Then cell survival was determined using Annexin V staining (left panel). Cell lysates were analyzed by Western blots using anti-Flag antibody to confirm doxycycline-

inducible expression (right panel). *$P < .05$; **$P < .01$, and ***$P < .001$.

Figure S4

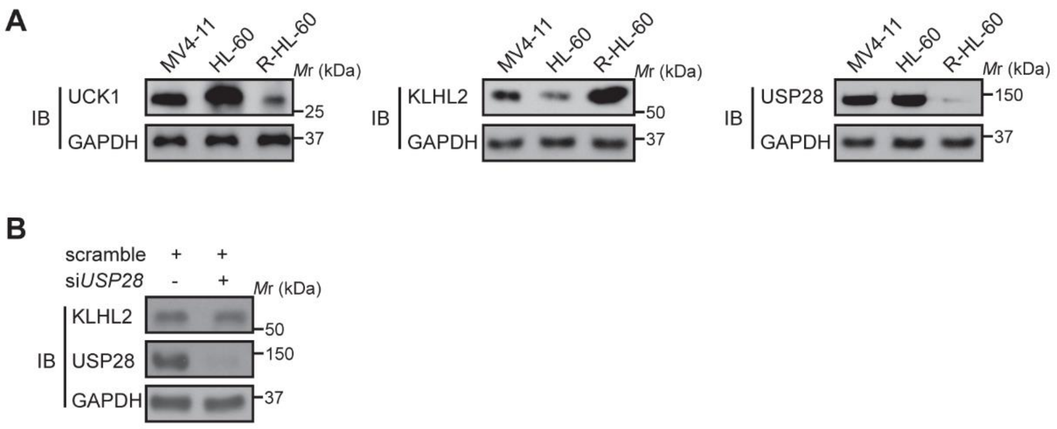

Figure S4. (**A**) The expression level of UCK1, KLHL2 and USP28 in MV4-11 and HL-60 cells. (**B**) USP28 knockdown didn't change the protein level of KLHL2. MV4-11 cells were transfected with or without USP28 siRNA, then immunoblotting was detected with indicated antibodies.

Figure S5

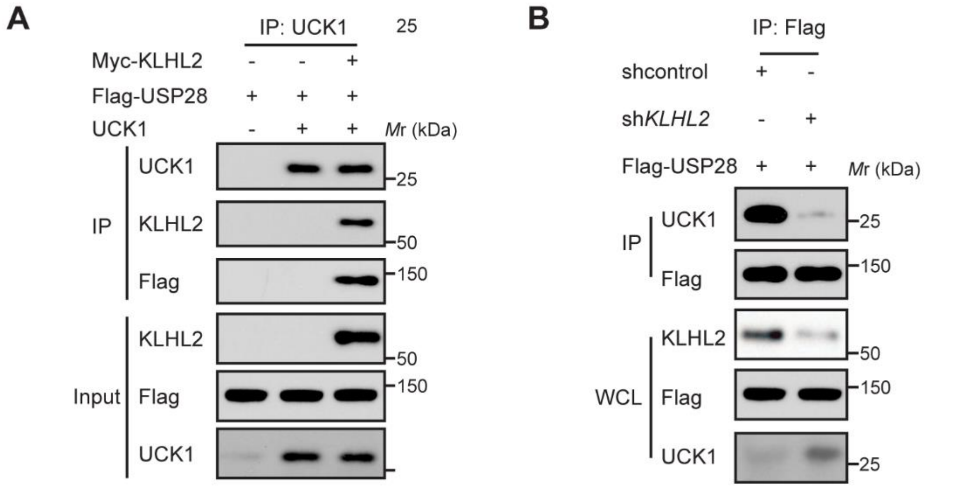

Figure S5. USP28 binds to UCK1 via KLHL2. (**A**) KLHL2 enhances binding of USP28 to UCK1. HEK293T cells were cotransfected with Flag-tagged USP28, UCK1, and Myc-KLHL2. 2 days later, cells were treated with the proteasome inhibitor MG132 for three hours and subjected to immunoprecipitation and immunoblotting analysis. (**B**) Silencing *KLHL2* decreases binding of USP28 to UCK1. HEK293T cells were cotransfected with Flag-USP28 and either shcontrol or KLHL2 shRNA-#1. After transfection, cells were lysed and immunoprecipitated with an anti-Flag antibody. Immunoblotting was performed with antibodies as indicated.

Figure S6

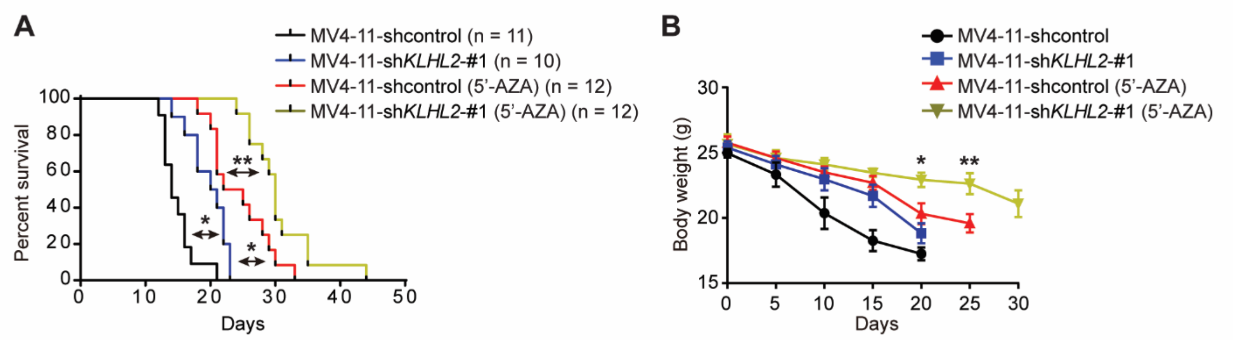

Figure S6. KLHL2 knockdown significantly sensitized 5'-AZA to inhibit MV4-11 cells *in vivo*. MV4-11 were divided into MV4-11-shcontro and MV4-11-sh*KLHL2*-#1. (**A**) Kaplan-Meier survival curves for recipients of MV4-11 cells with or without KLHL2 knockdown. (**E**) Body weight for AML mice with indicated treatment. Results were expressed as mean ± SEM of at least 10 mice in each group. *P < .05; **P < .01
